# Effects of *Funneliformis mosseae* on Growth and Photosynthetic Characteristics of *Camellia oleifera* under Different Nitrogen Forms

**DOI:** 10.3390/plants13030370

**Published:** 2024-01-26

**Authors:** Yuxuan Huang, Chuangxin Wang, Ziran Ma, Linping Zhang, Fei Wu

**Affiliations:** 1 College of Life Sciences, Northwest Normal University, Lanzhou 730070, China; 2 Key Laboratory of State Forestry and Grassland Administration on Forest Ecosystem Protection and Restoration of Poyang Lake Watershed, Jiangxi Agricultural University, Nanchang 330045, China

**Keywords:** arbuscular mycorrhizal fungi, *Camellia oleifera*, nitrogen fertilizer, photosynthesis

## Abstract

Nitrogen fertilizer increases agricultural yields but increases economic costs and causes a series of environmental problems. Arbuscular mycorrhizal fungi (AMF) have the potential to be used as biological fertilizer. However, the influence of nitrogen form on plant growth responsiveness to AMF inoculation is poorly understood. In this study, we investigated the effects of *Funneliformis mosseae* on growth, root morphology and photosynthetic characteristics of *Camellia oleifera* under different nitrogen forms during three harvest periods and clarified the most suitable nitrogen form for *C. oleifera*–AMF symbiosis. The results showed that urea, ammonium and nitrate nitrogen promoted plant growth and photosynthetic capacity, among which urea treatment had the highest value in all three harvests. No significant difference in plant growth parameters was observed between ammonium and nitrate nitrogen treatments in the first two harvests, while the plant height was significantly lower under ammonium nitrogen treatment than nitrate nitrogen treatment in the third harvest. Inoculation with *F*. *mosseae* in the presence of indigenous AMF could promote AMF colonization and plant growth at all three harvest times. Inoculation with *F*. *mosseae* significantly increased gas exchange parameters, the maximum photochemical efficiency (Fv/Fm) and the actual photochemical efficiency (ΦPSII). Inoculation with AMF increased the photochemical quenching coefficient (qP) better under urea treatment and improved the non-photochemical quenching coefficient (qN) better under ammonium nitrogen treatment. Principal component analysis showed that urea is the most beneficial nitrogen fertilizer for *C*. *oleifera*–AMF symbiosis. The results of this study provide a theoretical basis for the combination use of AMF and nitrogen fertilizer in agroforestry.

## 1. Introduction

Mineral elements such as nitrogen fertilizers are now the main source applicable to soils, and after the Green Revolution of the 1960s, nitrogen fertilizers synthesized through the Haber–Bosch process were widely used to increase different crop yields in agriculture and forestry [1]. The detrimental effects of nitrogen fertilizers are increasingly affecting human life, such as water entrophication, greenhouse gas emissions, etc. [1]. Nitrogen in soil exists mainly in both organic and inorganic forms, with inorganic nitrogen being more readily available for plant uptake and utilization, including ammonium (NH_4_^+^) and nitrate (NO_3_^−^) nitrogen. Different plants have preferences for different nitrogen forms. Some plants have a nitrate preference because nitrate is readily accessible for plants owing to its mobility, and at the same time, high concentrations of ammonium may have physiological toxicities on plants [2]. However, nitrate assimilation by plants uses more energy than ammonium assimilation; therefore, some plants show a preference for ammonium [3]. In addition, urea (CH_4_N_2_O) is the most commonly used nitrogen fertilizer because of its low cost, and when applied to the soil, urea is rapidly converted to ammonium and CO_2_ by microbial-derived urease [4]. Meanwhile, the assimilation and uptake pathways of ammonium nitrogen and urea are very similar; however, the toxicity of urea remains controversial [5,6]. It is important to reveal the preference of plants for nitrogen forms and how different nitrogen forms affect plant growth and photosynthetic characteristics, which will certainly help in the scientific application of nitrogen fertilizers.

*Camellia oleifera* Abel. (Theaceae), one of the world’s four famous woody oil plants, is an edible economic tree species unique to China [7]. *Camellia oleifera* has an unsaturated fatty acid content of up to 90%, much higher than that of vegetable oil, peanut oil and soybean oil, which has high economic and nutritional value [8]. With a history of more than 2300 years, *C*. *oleifera* is mainly distributed in red acidic soil areas in southern China, where nitrogen is the main limiting factor for its growth and yield [9]. How to improve the utilization efficiency of nitrogen fertilizer and ensure crop yield and quality while reducing the amount of nitrogen fertilizer used has been a concern of society and academia.

Arbuscular mycorrhizal fungi (AMF) are a class of soil microorganisms which can form a symbiosis with the roots of approximately 80% of land plants [10]. They can deliver mineral nutrients, especially nitrogen and phosphorus, to host plants and have garnered attention as potential biofertilizers [11,12]. AMF play an important role in soil nitrogen transformation and plant nitrogen uptake [13,14]. AMF can directly uptake nitrate, ammonium and small molecule organic nitrogen from the soil and translocate them to plants, enhancing the competitiveness of plant roots for nitrogen [15]. AMF have been reported to prefer ammonium over nitrate because ammonium is more energetically efficient [16], while some studies have found that AMF transfers higher amounts of nitrogen to plants when supplied with nitrate [17]. The preference of AMF for nitrogen forms and whether nitrogen forms alter the biological function of AMF on host plants are uncertain. A previous study showed that AMF can form a stable symbiotic relationship with *C*. *oleifera*, promoting its growth and nutrient acquisition [18]. In this study, we investigated the effects of AMF inoculation on *C*. *oleifera* growth, root morphology and photosynthetic characteristics under different nitrogen forms. Does the biological function of AMF change under different nitrogen forms? Does the *C*. *oleifera*–AMF symbiosis have a preference for nitrogen form? The objectives of this study are as follows: (1) to determine the effects of AMF supplementation on mycorrhizal colonization and biological function of AMF in unsterilized soil under different nitrogen forms and (2) to determine the optimal nitrogen form suitable for the growth of *C. oleifera*–AMF symbiosis The result may provide new insights into the use of AMF to enhance nitrogen fertilizer use efficiency.

## 2. Results

### 2.1. Mycorrhizal Colonization

**Figure 1:**
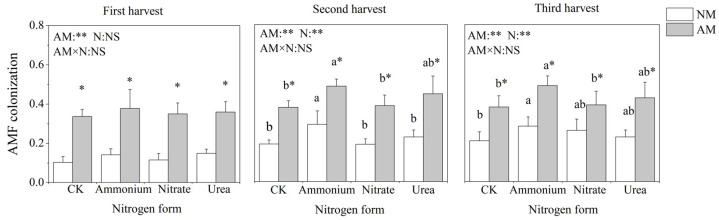
Effects of AMF inoculation and nitrogen application on the AMF colonization of *C*. *oleifera*. Note: Data are means ± SD (*n* = 5). Different asterisks indicate significant differences between different inoculation treatments under the same nitrogen level at *p* < 0.05; letters indicate significant differences in nitrogen levels under the same inoculation treatment at *p* < 0.05. * Significance level *p* < 0.05, ** significance level, and NS, no significant effect. N, nitrogen; NM, non-inoculated treatment; AM, inoculated treatment.

AMF colonization was observed in both inoculated and non-inoculated pots. Because the soil was not sterilized, some indigenous AMF were located in the soil, and mycorrhizal structures were also present in the uninoculated treatment. Inoculation with AMF significantly increased the AMF colonization rate of *C*. *oleifera* under each nitrogen treatment in the three harvests (Figure 1). Nitrogen application treatments significantly and positively affected AMF colonization except for the first harvest. The AMF colonization was highest in ammonium treatment, followed by urea, nitrate and CK treatment.

### 2.2. Plant Growth

Nitrogen addition significantly affected plant height and root morphology at the first harvest (Table 1). Urea addition significantly increased plant height by 42.67% under non-inoculated treatment, and the addition of ammonium nitrogen, nitrate nitrogen and urea increased plant height by 30.66%, 29.81% and 44.12%, respectively, under inoculated treatment. Nitrogen addition promoted root length, average diameter, surface area and volume, and the differences between the three nitrogen application treatments were not significant except for root surface area. Compared to ammonium and nitrate nitrogen, urea treatment significantly increased root surface area. Inoculation of AMF significantly affected root morphology of *C*. *oleifera* at first harvest. Inoculation of AMF increased root length by 11.62% to 18.65%.

Nitrogen application significantly and positively affected plant height, stem diameter and root morphology of *C*. *oleifera* at the second harvest (Table 2). Inoculation of AMF significantly affected plant height, root length, root average diameter, root volume and root surface area. The plant height of non-inoculated (20.77%, 22.13% and 32.79%) and inoculated plants (33.22%, 22.51% and 39.02%) under urea, nitrate and ammonium treatments was significantly higher than that under the no-nitrogen-added control. AMF inoculation increased plant height by 9.51% under no-nitrogen-application control. Inoculation of AMF did not significantly increase root length under ammonium and nitrate nitrogen treatments, whereas it increased root length by 8.56% under urea treatment.

Nitrogen application significantly increased plant height, stem diameter, root length, root average diameter, root volume and surface area in *C*. *oleifera* at the third harvest (Table 3). The plant height under urea and nitrate treatments was significantly higher than that under ammonium treatment. Inoculation of AMF significantly influenced plant height, stem diameter and root surface area. Under ammonium nitrogen treatment, inoculation with AMF increased plant height by 16.13% and stem diameter by 23.89%.

### 2.3. Gas Exchange Parameters

**Figure 2:**
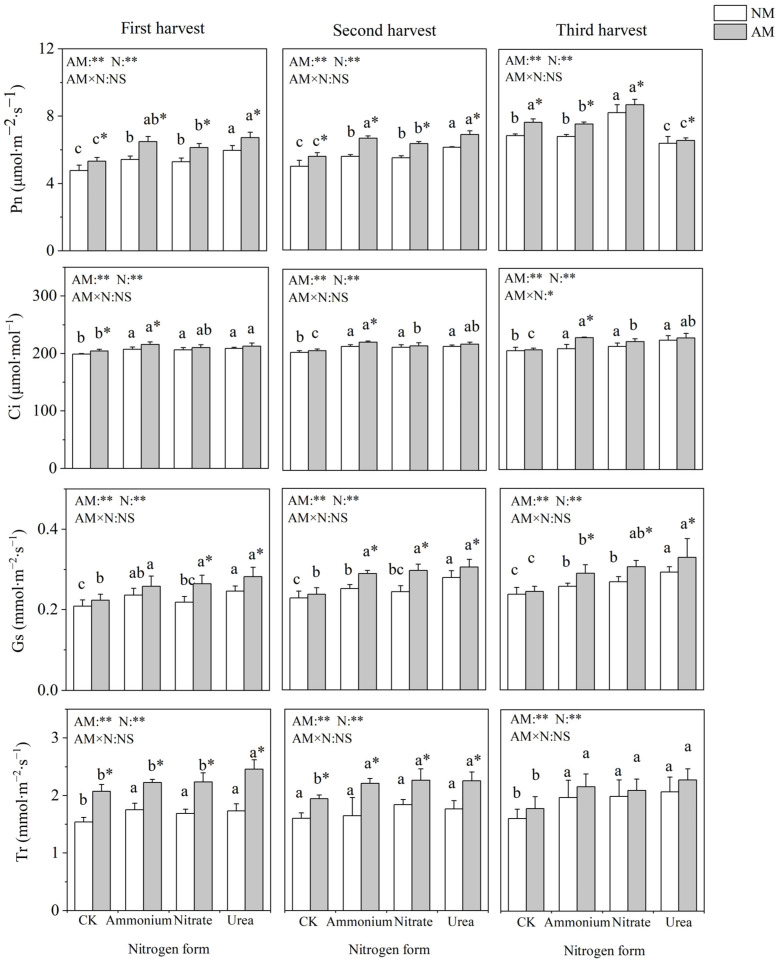
Effects of AMF inoculation and nitrogen application on gas exchange parameters of *C*. *oleifera*. Note: Data are means ± SD (*n* = 5). Different asterisks indicate significant differences between different inoculation treatments under the same nitrogen level at *p* < 0.05; letters indicate significant differences in nitrogen levels under the same inoculation treatment at *p* < 0.05. * Significance level *p* < 0.05, ** significance level, and NS, no significant effect. N, nitrogen; NM, no-inoculated treatment; AM, inoculated treatment.

Nitrogen application and inoculation with AMF significantly affected the gas exchange parameters of *C*. *oleifera* (Figure 2). Nitrogen addition significantly increased Pn, Gs, Ci and Tr, with the highest value observed under urea treatment. Pn was not significantly different between the nitrate and ammonium treatments. Inoculation with AMF significantly increased Pn under four nitrogen treatments and significantly increased Ci only under ammonium nitrogen treatment in all three harvests. Inoculation with AMF had no significant influence on Gs under the no-nitrogen-application control. In the absence of AMF inoculation, Tr did not differ significantly among the different nitrogen form treatments, and when inoculated with AMF, the different nitrogen forms significantly affected Tr in the first and third harvest (fall raw growing seasons).

### 2.4. Chlorophyll Fluorescence Parameters

**Figure 3:**
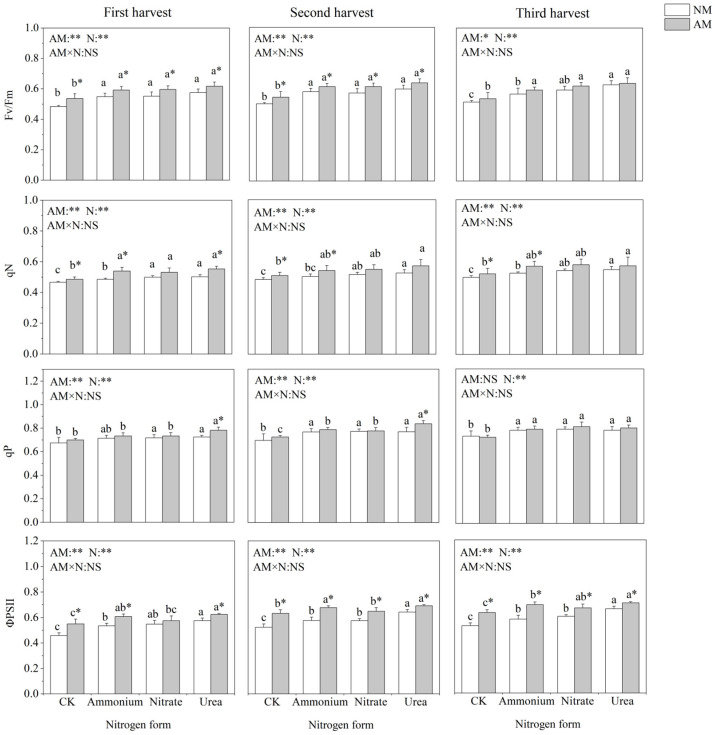
Effects of AMF inoculation and nitrogen application on chlorophyll fluorescence parameters of *C*. *oleifera*. Note: Data are means ± SD (*n* = 5). Different asterisks indicate significant differences between different inoculation treatments under the same nitrogen level at *p* < 0.05; letters indicate significant differences in nitrogen levels under the same inoculation treatment at *p* < 0.05. * Significance level *p* < 0.05, ** significance level, and NS, no significant effect. N, nitrogen; NM, non-inoculated treatment; AM, inoculated treatment.

Nitrogen application and inoculation with AMF significantly affected chlorophyll fluorescence parameters of *C*. *oleifera* (Figure 3). Nitrogen addition significantly increased Fv/Fm, qN, qP and ΦPSII. Fv/Fm and qN did not differ significantly between the three nitrogen addition treatments. In the absence of AMF inoculation, qP did not differ significantly among the different nitrogen forms, and when inoculated with AMF, the different nitrogen forms significantly affected qP in the three harvest times. Inoculation with AMF significantly increased Fv/Fm and ΦPSII in all three harvests. Under urea treatment, inoculation with AMF could significantly increase qP. Under ammonium treatment, inoculation with AMF significantly increased qN.

### 2.5. The Results of PCA

In order to screen the optimal nitrogen form and the role of AMF inoculation in the growth of oil tea, the effects of nitrogen form were comprehensively evaluated by PCA. Fourteen indicators, height, stem diameter, root length, root diameter, root volume, root surface area, Pn, Ci, Tr, Gs, Fv/Fm, ΦPSII, qP and qN, were used as raw indicators, and the data were standardized for PCA.

Pn, root length and qP play a major role for the first principal component and stem diameter for the second principal component (Table 4). Based on the eigenvalues and factor loading matrices of the two principal components, the computational common for each principal score is derived as follows:

*Y*1 = 0.294*X*1 + 0.253*X*2 + 0.301*X*3 + 0.179*X*4 + 0.261*X*5 + 0.281*X*6 + 0.308*X*7 + 0.295*X*8 + 0.292*X*9-0.034*X*10 + 0.278*X*11 + 0.230*X*12 + 0.269*X*13 + 0.276*X*14

*Y*2 = 0.303*X*1 + 0.461*X*2 + 0.265*X*3 − 0.451*X*4-0.335*X*5-0.327*X*6 + 0.226*X*7 − 0.036*X*8 + 0.014*X*9 + 0.263*X*10 − 0.270*X*11 + 0.043*X*12 − 0.076*X*13 + 0.006*X*14

The composite scores and ranking of the available main components were calculated (Table 5). The results showed that regardless of AMF inoculation, nitrogen application treatments showed urea > nitrate > ammonium > CK. Under the same nitrogen treatment: AM > NM.

## 3. Discussion

### 3.1. AMF Colonization and Plant Growth

AMF is a type of specialized living nutrient symbiotic microorganisms, and AMF colonization of the root system is a prerequisite for its functioning [19]. This study was conducted in non-sterilized soil, mycorrhizal structures could be seen even in the absence of AMF inoculation, and AMF addition promoted mycorrhizal formation in *C*. *oleifera*. A similar finding was observed in *Populus* × *canadensis* ‘Neva’ by Liu et al. [20]. AMF can access both ammonium and nitrate nitrogen to regulate their reproduction and colonization [14]. In this study, ammonium nitrogen significantly improved AMF colonization compared to nitrate nitrogen. Some studies suggested that AMF prefer ammonium relative to nitrate because ammonium is energetically more efficient [21,22]. The results of the present study were consistent with this hypothesis.

The ability of AMF to promote the growth of host plants has been demonstrated in a number of cash crops, such as maize [23] and citrus [24]. Most of the previous studies were performed under sterilized soils. However, indigenous AMF and other soil microorganisms in rhizosphere soil may influence the interaction between AMF and host plants and thus affect their biological functions [25]. Liu et al. [20] showed that AM symbiosis increased the biomass parameters, such as stem length, ground diameter, dry weight, chlorophyll content and gas exchange capacity, of *Populus* × *canadensis* ‘Neva’ in both sterilized and unsterilized soil. However, Ndoye et al. [26] showed that the promotion of *Acacia senegal* (L.) Willd. growth by inoculation of AMF in unsterilized soil was not significant due to AMF colonization already present in the unsterilized soil. In the present study, when indigenous AMF was present, AMF addition in the first harvest had no significant effect on plant height and ground diameter but promoted root growth. In the second harvest, AMF addition promoted plant height and root growth, and in the third harvest, it promoted mainly plant height and ground diameter and had little effect on root growth. This indicated that *F*. *mosseae* mainly promoted root growth of *C. oleifera* seedlings in the early period of AMF inoculation and mainly promoted the growth of aboveground in the later period of AMF inoculation. Previous studies found that plant responsiveness to AMF was influenced by genotype, environmental conditions, etc. [27]. Some plant–AMF combinations increase shoot growth, while others promote root growth better [27]. The results of this study showed that the response of *C*. *oleifera* to AMF was related to the growth stage. *Funneliformis mosseae* may first improve root growth to increase nutrient absorption and then promote aboveground growth of *C*. *oleifera*.

Soil nitrogen status is another factor that may affect plant response to AMF. In all three harvests, nitrogen addition promoted root and aboveground growth regardless of nitrogen forms, but the promotion degree was different. The plant height and root growth of urea treatment were the highest among three nitrogen forms in all three harvests. No significant difference was observed in plant growth parameters between ammonium and nitrate treatments in the first two harvests, while the plant height was significantly lower under ammonium treatment than nitrate treatment in the third harvest. This may result from ammonium toxicity caused by the long-term single application of ammonium nitrogen [28]. The research on the effect of AM fungi on plant growth under different nitrogen forms is limited. Song et al. [29] showed that the effect of AMF on the growth of the invasive plant *Solidago canadensis* was independent of nitrogen forms, whilst AMF eliminates the influence of nitrogen forms on native plants. In this study, the effect of AMF was different in different nitrogen forms. In the first and second harvests, the positive effect of AMF on root length under urea treatment was better than that of other nitrogen form treatments, while in the third harvest, the promoting effect of AMF on plant height under ammonium nitrogen treatment was higher than that of other nitrogen form treatments. Previous studies reported that AMF preferred to obtain ammonium in comparison to nitrate because nitrate needs to be reduced to ammonium in hyphae before it can be assimilated, a process that requires more energy [16]. Ammonia/ammonium generated by the hydrolysis of urea is the main form of urea utilization by plants, which may be the reason why AMF has a positive effect on plant growth under urea treatment [30]. The plant height in inoculated plants was significantly higher than that of non-inoculated plants in the third harvest, which is probably due to the inoculated *F*. *mosseae* promoting the mycorrhizal pathway to absorb nitrogen and alleviate the ammonium toxicity of long-term ammonium nitrogen application to *C*. *oleifera* [31].

### 3.2. Funneliformis mosseae Improved Photosynthetic Characteristics of C. oleifera

Photosynthesis is the material basis of plant growth and development, which directly affects the quality and yield of *C*. *oleifera* [18,32]. It is well known that nitrogen is a vital element of chlorophyll and is essential for plant photosynthesis [33]. In line with a previous study on *C*. *oleifera*, nitrate and ammonium treatments showed a similar promoting effect on Pn [33]. Urea addition significantly increased Pn compared with nitrate and ammonium nitrogen, which was consistent with plant growth parameters, and supported the important role of photosynthesis in plant growth [32]. Many woody plants that form symbiosis with AMF can effectively increase the photosynthetic rate and promote plant growth and development [34]. In this study, inoculation with AMF significantly increased almost all aspects of photosynthesis, suggesting that AMF could enhance the photosynthetic capacity of the host, thus obtaining the photosynthate needed for its own growth from the host [35]. Previous studies have shown that AMF improves the photosynthetic characteristics of host plants [36], and the results of the present study indicated that in the presence of indigenous AMF, the improvement of photosynthetic characteristics by AMF inoculation remained efficient. Enhanced photosynthetic characteristics of AMF-colonized *C*. *oleifera* seedlings may be due to three reasons. Firstly, AMF colonization may affect hormone (i.e., abscisic acid [ABA]) levels of host plants that regulate stomatal conductance, thus impacting photosynthetic efficiency [37]; and secondly, AMF colonization also enhances water uptake capacity by improving the transpiration rate, thus increasing the photosynthetic rate [36]. Thirdly, AMF may enhance carbohydrate cunning and stimulate the photosynthetic rate to replace the carbon requirement of AMF [38]. Different nitrogen forms may affect the photosynthetic properties of plants and the symbiotic relationship between AMF and host plants. Interestingly, inoculation with AMF increased qP better under urea treatment and improved qN better under ammonium treatment. The qP represents the part of light energy used to drive photosynthesis [39]. An increase in qP indicated a more efficient utilization of light by AMF under urea treatment. The qN represents the part of light energy received by PSII that is released by heat, and this process protects the photosynthetic machine from excessive light [39]. The higher qN in inoculated plants than that of non-inoculated plants under ammonium treatment may protect the photosynthetic apparatus under ammonium toxicity conditions, thus promoting photosynthesis and plant growth.

### 3.3. Optimal Nitrogen Form of C. oleifera–AMF Symbiosis

In this study, nitrogen and AMF could improve the growth and photosynthetic characteristics of *C*. *oleifera*. In order to further investigate the effects of nitrogen form on the biological function of AMF and to clarify which nitrogen form had the best effect on *C*. *oleifera*–AMF symbiosis, PCA was used to rank the advantages and disadvantages. The results showed that urea had the best effect on *C*. *oleifera*–AMF symbiosis promotion, regardless of whether AMF was added or not. This may be due to the fact that urea can be hydrolyzed to produce CO_2_ and increased soil carbon to avoid soil C:N imbalance due to nitrogen fertilization. It is also interesting to note that while the addition of ammonium nitrogen significantly promoted AMF colonization, nitrate nitrogen, however, was ranked superior to ammonium nitrogen, although it was shown that AMF mycelium uptake of ^15^NH_4_^+^ (per unit weight) was 15 times higher than ^15^NO_3_^−^ uptake (per unit weight), and the rate of uptake of ^15^NH_4_^+^ was higher than that of ^15^NO_3_^−^ [40]. However, charge imbalance problems may not be encountered when the form of nitrogen acquisition is primarily nitrate. Plant uptake of nitrate and cations such as K^+^ and Ca^+^ from the soil avoids changes in the electrochemical potentials of the exchange surfaces. At the same time, ammonium uptake requires proton secretion (or anion uptake), which may alter soil pH and make further ammonium uptake more difficult [41].

## 4. Materials and Methods

### 4.1. Biological Materials and Soil

The seeds of *C*. *oleifera* were provided by the Jiangxi Academy of Forestry Research, China. The seeds were surface sterilized and cleaned with potassium permanganate and sterile water first, germinated on wet sterilized sand, and finally uniform seedlings were selected and removed to pots.

The AMF strain (*Funneliformis mosseae*) was provided by Professor Qiangsheng Wu, Institute of Root Biology, Yangtze University, Hubei, PR China. The inoculum of *F*. *mosseae* was propagated on white clover roots and consisted of spores (34 ind.·g^−1^), root fragments, mycelium and soil.

The growth medium used in this experiment contained soil and sand (1:1 *v*/*v*). The soil (pH = 5.8) was collected from Jiangxi Agricultural University (Nanchang, China) and had the following physicochemical properties: available P, 2.30 mg·kg^−1^; NH_4_^+^-N, 8.75 mg·kg^−1^; NO_3_^−^-N, 2.04 mg·kg^−1^; organic matter, 22.35 g·kg^−1^; and pH 6.2. The sand was washed with tap water until the water ran clear.

### 4.2. Experimental Design

The experimental layout had two factors: (i) the AMF inoculation treatment, either inoculation with *F*. *mosseae* (AM) or the non-inoculated control (NM); and (ii) the nitrogen application treatment with NH₄HCO₃ (ammonium), Ca(NO_3_)_2_ (nitrate), CO(NH_2_)_2_ (urea) and no-nitrogen-added controls (CK). Each treatment had 30 replicates. The seedlings were planted in pots (14.5 cm × 15 cm) filled with 1.5 kg of growth substrate. Each pot was inoculated with 60 g of inoculum (*n* = 30), and the other pots (NM controls; *n* = 30) received 60 g of autoclaved inoculum plus 10 mL of inoculum filtrate to provide a typical microbial population free of AMF propagules.

One seedling was implanted in each pot, ensuring that the root was in contact with the AMF. The experiment was conducted in a plastic greenhouse (temperature: 10 to 30 °C; humidity: 30 to 60%) under natural light at Jiangxi Agricultural University from May 2021 to September 2022. In addition, at the beginning of the second year of the experiment (March 2022), we made a second addition of AMF.

Before nitrogen application, 200 mL of 1/4 Hoagland nutrient solution was watered each month. After 3 months of growth, nitrogen treatment started to be applied. A solution of 250 mL of water containing 5 mM of NH_4_HCO_3_, 2.5 mM of Ca(NO_3_)_2_ and 2.5 mM of CO(NH_2_)_2_ with 1/4 nitrogen-free Hoagland’s nutrient solution was watered at 7 d intervals for a total of 10 waterings and then incubated for 1 week before the 1st harvest in October 2021 (fall growing season). Nitrogen treatments were applied once during winter dormancy from November 2021 to January 2022. Subsequent nitrogen treatments were applied every 14 d with 1/4 nitrogen-free Hoagland nutrient solution for a total of eight nitrogen application treatments. The 2nd collection was made in June 2022 (summer growing season). After the 2nd harvest was completed, the nitrogen treatments described above were repeated every 14 days for a total of eight application treatments. The 3rd collection of samples was in September 2022 (fall growing season). Gas exchange parameters and chlorophyll fluorescence parameters were measured before sample harvesting.

### 4.3. Mycorrhizal Colonization

Roots were washed at harvest to take fresh plant samples for mycorrhizal colonization measurements. The method of mycorrhizal colonization described by Phillips and Hayman [42]. Mycorrhizal colonization rate was determined by using the gridline intersection method under a light microscope [43].

### 4.4. Plant Growth

Plant height was measure using a measuring tape (Deli, Nanchang, China). Ground diameter was measured using vernier calipers (Deli, Nanchang, China). The overall root system of *C. oleifera* seedlings was scanned using Espon Perfection V700 Photo type scanner (Seiko Epson Corporation, Shiojiri, Japan), and root morphology was analyzed using WinRHIZO (Pro2012) root software.

### 4.5. Measurement of Gas Exchange Parameters and Chlorophyll Fluorescence Parameters

Three healthy leaves (3rd to 5th under the terminal shoot) were selected from each oil tea plant, and the net photosynthetic rate (Pn), stomatal conductance (Gs), intercellular CO_2_ concentration (Ci) and transpiration rate (Tr) were measured using the LI-6400 Portable Photosynthesizer (LI-COR Inc., Lincoln, NE, USA) at 9:00 a.m. before each harvest time.

Before measuring the chlorophyll fluorescence parameters, the plants were dark-treated for 30 min, and three complete unfolded leaves were selected from each oil tea plant to determine the maximum photochemical efficiency (Fv/Fm), the actual photochemical efficiency (ΦPSII), the non-photochemical burst (qN) and the photochemical burst (qP), using the PAM-2500 Portable Modulated Chlorophyll Fluorescence Meter (Walz Inc., Nuremberg, Germany).

### 4.6. Data Analysis

Statistical analyses were performed with SPSS software, version 20.0 (SPSS Inc., Chicago, IL, USA). The data were checked for normality and homogeneity of variances using the Kolmogorov–Smirnov test and Levene test, respectively. A two-way ANOVA was performed to examine the significance of AMF inoculation, nitrogen application treatment and their interaction. A one-way ANOVA was used to test the differences among the different inoculation treatments under the same nitrogen treatment. Means were compared with Duncan’s multiple range test at the 5% level. Combining the data from three sample collections, principal component analysis (PCA) was used to comprehensively evaluate and select the best nitrogen form most suitable for the symbiotic growth of *C*. *oleifera*–AMF.

## 5. Conclusions

*Funneliformis mosseae* can promote the growth, root formation and photosynthesis capacity of *C. oleifera*. Urea nitrogen fertilizer is the most beneficial nitrogen fertilizer for *C*. *oleifera* growth and photosynthesis. Compared with ammonium nitrogen, nitrate nitrogen was more effective in promoting *C*. *oleifera*–AMF symbiosis. The results of this experiment can provide a theoretical basis for the subsequent research and development of AMF to improve the efficiency of nitrogen fertilizer utilization in agriculture.

## Figures and Tables

**Table 1:** Effects of AMF inoculation and nitrogen application on the plant growth and root morphology of *C*. *oleifera* at first harvest.

AM Treatment	N Treatment	Height (cm)	Stem Diameter (cm)	Root Length (cm)	Root Average Diameter (cm)	Root Volume (cm^3^)	Root Surface Area (cm^2^)
NM	CK	11.06 ± 1.62 b	2.6 ± 0.31	127.86 ± 11.72 b	0.65 ± 0.11 b	0.73 ± 0.11 b	51.03 ± 2.34 c
	Ammonium	13.72 ± 2.03 ab	2.81 ± 0.26	148.49 ± 14.25 a	0.84 ± 0.13 a	1.11 ± 0.1 a	66 ± 4.35 b
	Nitrate	13.44 ± 2.63 ab	2.8 ± 0.36	153.37 ± 20.08 a	0.98 ± 0.08 a	1.17 ± 0.07 a	67.45 ± 5.85 b
	Urea	15.78 ± 2.43 a	2.88 ± 0.49	162.07 ± 13.56 a	0.85 ± 0.15 a	1.18 ± 0.05 a	78.35 ± 4.72 a
AM	CK	11.74 ± 1.96 b	2.8 ± 0.31	151.7 ± 10.3b *	0.73 ± 0.12 b	0.85 ± 0.05 c *	53.76 ± 2.34 c
	Ammonium	15.34 ± 2.05 a	2.94 ± 0.23	165.75 ± 16.31 ab	0.94 ± 0.19 a	1.27 ± 0.17 b	77.67 ± 6.19 b *
	Nitrate	15.24 ± 1.93 a	2.86 ± 0.22	178.94 ± 24.36 a	1.02 ± 0.15 a	1.37 ± 0.14 ab *	76.25 ± 5.48 b *
	Urea	16.92 ± 2.07 a	2.87 ± 0.37	186.89 ± 11.86 a *	0.98 ± 0.12 a	1.47 ± 0.17 a *	87.91 ± 5.12 a *
Two-way ANOVA							
N		9.417 **	0.661 NS	8.696 **	9.380 **	42.778 **	72.016 **
AM		3.853 NS	0.854 NS	20.257 **	4.447 *	27.163 **	29.631 **
N × AM		0.143 NS	0.189 NS	0.143 NS	0.198 NS	0.999 NS	1.626 NS

Note: Data are means ± SD (*n* = 5). Different asterisks indicate significant differences between different inoculation treatments under the same nitrogen level at *p* < 0.05; letters indicate significant differences in nitrogen levels under the same inoculation treatment at *p* < 0.05. * Significance level *p* < 0.05, ** significance level, and NS, no significant effect. N, nitrogen; NM, non-inoculated treatment; AM, inoculated treatment.

**Table 2:** Effects of AMF inoculation and nitrogen application on the plant growth and root morphology of *C*. *oleifera* at second harvest.

AM Treatment	N Treatment	Height (cm)	Stem Diameter (mm)	Root Length (cm)	Root Average Diameter (cm)	Root Volume (cm^3^)	Root Surface Area (cm^2^)
NM	CK	18.3 ± 0.58 c	3.92 ± 0.15 b	186.83 ± 9.01 d	0.7 ± 0.11 c	1.03 ± 0.07 c	54.25 ± 2.09 c
	Ammonium	22.1 ± 1.6 b	4.2 ± 0.19 b	216.23 ± 9.84 c	0.92 ± 0.12 ab	1.23 ± 0.04 b	72.11 ± 9.21 b
	Nitrate	22.35 ± 0.47 b	4.12 ± 0.13 b	221.62 ± 10.62 b	1.04 ± 0.07 a	1.31 ± 0.11 ab	74.37 ± 9.17 ab
	Urea	24.3 ± 1.48 a	4.66 ± 0.43 a	262.16 ± 9.22 a	0.82 ± 0.05 b	1.39 ± 0.13 a	83.28 ± 3.88 a
AM	CK	20.04 ± 0.84 c *	3.98 ± 0.18 b	197.79 ± 3.66 c *	0.73 ± 0.1 c	1.04 ± 0.11 b	56.87 ± 1.96 c
	Ammonium	24.38 ± 1.84 ab	4.14 ± 0.11 b	220.19 ± 9.26 b	0.9 ± 0.16 b	1.33 ± 0.16 a	81.48 ± 3.81 b
	Nitrate	22.42 ± 1.95 b	4.25 ± 0.33 b	233.58 ± 15.6 b	1.04 ± 0.14 b	1.3 ± 0.08 a	79.17 ± 4.91 b
	Urea	25.44 ± 2.04 a	4.33 ± 0.33 a	284.6 ± 10.7 a *	1.01 ± 0.14 a *	1.42 ± 0.1 6a	87.4 ± 4.99 a
Two-way ANOVA							
N		26.455 **	8.898 **	109.797 **	13.511 **	18.508 **	50.408 **
AM		7.857 **	0.494 NS	14.606 **	1.842 *	0.841 **	8.538 **
N × AM		1.029 NS	1.863 NS	1.395 NS	1.600 NS	0.361 NS	0.663 NS

Note: Data are means ± SD (*n* = 5). Different asterisks indicate significant differences between different inoculation treatments under the same nitrogen level at *p* < 0.05; letters indicate significant differences in nitrogen levels under the same inoculation treatment at *p* < 0.05. * Significance level *p* < 0.05, ** significance level, and NS, no significant effect. N, nitrogen; NM, non-inoculated treatment; AM, inoculated treatment.

**Table 3:** Effects of AMF inoculation and nitrogen application on the plant growth and root morphology of *C*. *oleifera* at third harvest.

AM Treatment	N Treatment	Height (cm)	Stem Diameter (cm)	Root Length (cm)	Root Average Diameter (cm)	Root Volume (cm^3^)	Root Surface Area (cm^2^)
NM	CK	20.06 ± 2.65 c	4.8 ± 0.46 b	207.89 ± 11.06 c	0.72 ± 0.11 c	1.04 ± 0.07 c	56.75±1.02 c
	Ammonium	24.42 ± 1.77 b	4.52 ± 0.54 b	238.34 ± 3.84 b	0.93 ± 0.12 ab	1.24 ± 0.04 b	74.32±9.2 b
	Nitrate	31 ± 3.8 a	4.82 ± 0.39 b	261.44 ± 10.6 b	1.03 ± 0.04 a	1.31 ± 0.11 ab	76.57±9.11 b
	Urea	32.1 ± 3.57 a	5.62 ± 0.48 a	280.67 ± 9.97 a	0.89 ± 0.07 b	1.39 ± 0.13 a	85.21±3.57 a
AM	CK	19.92 ± 2.36 c	4.84 ± 0.17 b	209.89 ± 2.59 c	0.74 ± 0.10 b	1.04 ± 0.11 b	57.36±3.31 c
	Ammonium	28.36 ± 2.36b *	5.6 ± 0.50a *	236.82 ± 8.01 b	0.91 ± 0.16 ab	1.33 ± 0.16 a	82.86±3.77 b
	Nitrate	32.9 ± 1.75 a	5.56 ± 0.48 a *	249.61 ± 14.57 b *	1.05 ± 0.15 a	1.3 ± 0.08 a	80.5±5.18 b
	Urea	33.84 ± 2.97 a	5.96 ± 0.43 a	302.47 ± 10.7 a *	1.01 ± 0.14 a *	1.42 ± 0.16 a	89.63±4.99 a
Two-way ANOVA							
N		47.244 **	8.630 **	128.236 **	12.816 **	18.338 **	51.706 **
AM		4.585 *	15.316 **	0.731 NS	1.205 NS	0.734 NS	5.918 *
N × AM		0.921 NS	2.621 NS	5.308 **	0.740 NS	0.343 NS	0.819 NS

Note: Data are means ± SD (*n* = 5). Different asterisks indicate significant differences between different inoculation treatments under the same nitrogen level at *p* < 0.05; letters indicate significant differences in nitrogen levels under the same inoculation treatment at *p* < 0.05. * Significance level *p* < 0.05, ** significance level, and NS, no significant effect. N, nitrogen; NM, non-inoculated treatment; AM, inoculated treatment.

**Table 4:** Principal component analysis factor load matrix.

Index	Principal Component 1	Principal Component 2
Height (*X*1)	0.850	0.367
Stem diameter (*X*2)	0.732	0.558
Root length (*X*3)	0.869	0.321
Root average diameter (*X*4)	0.514	−0.546
Root volume (*X*5)	0.753	−0.405
Root surface area (*X*6)	0.811	−0.396
Pn (*X*7)	0.89	0.273
Gs (*X*8)	0.852	−0.043
Ci (*X*9)	0.844	0.017
Tr (*X*10)	−0.101	0.318
Fv/Fm (*X*11)	0.802	−0.327
qP (*X*12)	0.866	0.052
qP (*X*13)	0.776	−0.092
qN (*X*13)	0.776	0.008
ΦPSⅡ (*X*14)	0.798	0.367

**Table 5:** Principal component score and ranking of AM treatment and nitrogen treatment.

AM Treatment	N Treatment	*Y*1	*Y*2	Comprehensive Score	Rank
NM	CK	128.486	92.156	8168.572	7
	Ammonium	151.076	48.249	8176.879	6
	Nitrate	155.811	51.355	8471.011	5
	Urea	167.637	56.171	9135.742	2
AM	CK	140.568	47.017	7658.589	8
	Ammonium	160.421	48.937	8628.098	4
	Nitrate	162.188	52.896	8804.381	3
	Urea	178.048	61.283	9741.72	1

## Data Availability

All data are included in the main text.
